# Teneurin C-terminal associated peptides (TCAP): modulators of corticotropin-releasing factor (CRF) physiology and behavior

**DOI:** 10.3389/fnins.2013.00166

**Published:** 2013-09-17

**Authors:** Yani Chen, Mei Xu, Reuben De Almeida, David A. Lovejoy

**Affiliations:** Department of Cell and Systems Biology, University of TorontoToronto, ON, Canada

**Keywords:** stress, HPA axis, glucocorticoids, dystroglycan, ERK1/2, cytoskeleton, peptide evolution

## Abstract

The existence of the teneurin C-terminal associated peptides (TCAP) was reported in 2004 after screening a rainbow trout hypothalamic cDNA for corticotropin-releasing factor (CRF)-related homologs. In vertebrates, there are four TCAP paralogs, where each peptide is associated with a teneurin transmembrane protein. The TCAPs are 40 or 41 amino acids in length and possess less than 20% residue identity with the CRF family of paralogs. Orthologs of TCAP are found in all metazoans with the possible exception of poriferans and cnidarians. Recent evidence indicates that TCAP and the teneurins may have been introduced into the Metazoa via horizontal gene transfer from prokaryotes into a basal protistan. Thus, the origin of the TCAPs likely predated that of the CRF family. In the mammalian brain, TCAP-1 is transcribed independently from teneurin-1. Moreover, TCAP-1 acts on neurons by a CRF-receptor independent signal transduction pathway to regulate cellular cytoskeletal function to stimulate cell activity. Administration of synthetic TCAP-1 to rodents inhibits a number of CRF- and stress-associated behaviors via a hypothalamic–pituitary–adrenal (HPA) axis-independent mechanism.

## Introduction

Corticotropin-releasing factor (CRF) is part of an evolutionary conserved peptide family integral to the regulation of stress-associated behavior and physiology in metazoans. The long period of time during which CRF-like peptides have persisted suggests that the CRF response has been evolutionarily advantageous. Early-evolving gene systems integral to the survival of an organism are evolutionarily selected for, and because of this, may give rise to additional paralogous lineages via gene, genomic, and chromosomal duplication events. In addition, such early evolving systems will likely become associated with newer evolving molecular and physiological systems. Thus, because of the evolutionary age of the CRF system, it is likely that it is modulated, in turn, by even earlier evolving systems.

One such candidate for an early evolving CRF-modulatory system may be represented by TCAP-1, a member of the teneurin C-terminal-associated peptides family. Highly conserved in all metazoans, TCAP-1 is active in the regulation of metabolism, stress, and reproduction and significantly inhibits a number of CRF-induced stress responses. Although TCAP was originally identified in a rainbow trout cDNA library screen for potential CRF homologs and share a number of structural similarities with the CRF family of peptides, they have less than 20% sequence similarity to the CRF family (Qian et al., 2004; Lovejoy et al., 2006). *In situ* experiments in Sprague-Dawley rats show that TCAP is highly expressed in all hippocampal subregions, central amygdala (CeA), basolateral amygdala (BLA), and various hypothalamic nuclei regions that are known to express CRF receptors (Wang et al., 2005). The TCAP-1 peptide also blocks both CRF-induced stress behaviors and c-fos activation within brain areas known to modulate behavioral responses to stress in Wistar rats (Tan et al., 2009). Recent studies show that TCAP exerts its neuromodulatory role on CRF elements independent of the hypothalamic–pituitary–adrenal (HPA) axis, harboring the idea that several accessory pathways may have evolved alongside the HPA axis-mediated signaling pathway.

## CRF evolution and physiology

**Figure 1:**
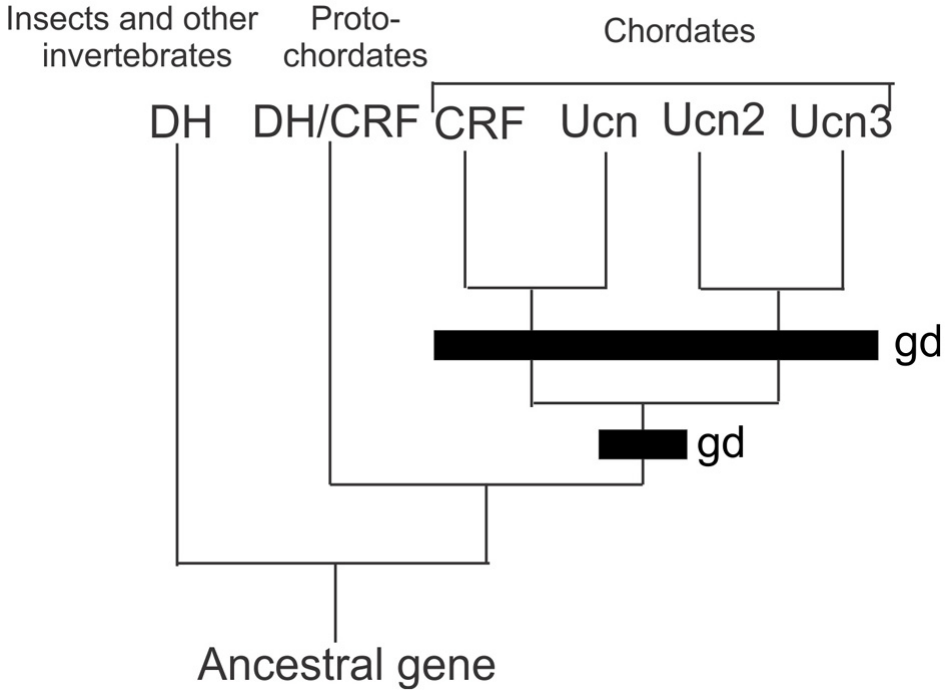
**Comparison of the CRF peptides family in chordates by genome duplication.** Two rounds of genome duplications early in chordate ancestry gave rise to the four paralogous CRF related peptides. Abbreviations: DH, diuretic hormone; DH/CRF, diuretic hormone/corticotrophin releasing factor like peptide; CRF, corticotrophin releasing factor; UCN, urocortin; UCN2, urocortin 2; UCN3 urocortin 3; gd, genome duplication event.

In vertebrates (Mammalia, Amphibia, Actinoptergyii, and Chondrichthyes), the CRF family consists of four paralogous peptides that are the result of two rounds of genome duplications in early evolution and has been previously described in detail (Lovejoy and Balment, 1999; Lovejoy and Jahan, 2006) (Figure 1). Orthologs of CRF in invertebrates occur as the diuretic hormones in arthropods, and diuretic hormone-like peptide in tunicates. These findings indicate that only a single CRF peptide was present in the genome of an ancestral protochordate. The first of the genome duplications led to the formation of two CRF-related peptides. After subsequent selection, both lineages were retained but modified during the accrual of mutations leading to a modified amino acid sequence. One peptide shares sequence similarities to both CRF and urotensin-1 (urocortin in mammals; Vaughan et al., 1995; Donaldson et al., 1996), whereas the second peptide possesses both urocortin 2 and 3 characteristics (Lovejoy and Jahan, 2006). The second genome duplication led to the formation of the four peptides that are found in extant vertebrates (Lewis et al., 2001; Reyes et al., 2001). However, the presence of CRF orthologs in arthropods indicates that the earliest CRF-like peptides existed before the bifurcation of deuterostome and protostome animals (Lovejoy and Balment, 1999; Lovejoy and Jahan, 2006). Research in the last few decades has provided a basis for understanding the regulation of stress and metabolism by CRF. Currently, it is understood that in vertebrates, CRF activates the stress response through the HPA axis. It is also through this pathway that CRF is regulated by modulatory components of the central nervous system.

The ancient evolution of CRF and CRF-like peptides indicates that there is functional necessity of the actions of CRF on regulatory mechanisms in physiology. In many species, humans included, CRF induces behaviors and physiological adaptations that endeavor to cope with environmental stressors. Stressors that are associated with new environments, for example, generally bring about behaviors of increased arousal and decreased food intake. Changes in behaviors and physiology that are induced by CRF are mediated by interactions of CRF/Ucn with both the CRF_1_ and CRF_2_ receptors. Urocortin-2 and -3 mediate their functions through a separate CRF_2_ receptor (Hauger et al., 2006).

In numerous vertebrates including Osteichthyes, Amphibia, Reptilia, Avia, and Mammalia species, exposure to stress leads to activation of the hypothalamic–pituitary–adrenocortical (HPA) axis (Barsyte et al., 1999; Lovejoy and Balment, 1999; Lovejoy and Jahan, 2006; Denver, 2009; Lovejoy, 2009). The HPA axis is a neuroendocrine feedback system that is activated when afferents from the sensory system and brainstem signal to the paraventricular nucleus (PVN) to secrete CRF and arginine vasopressin (AVP) hormones into the hypophyseal portal system (Tsigos and Chrousos, 2002; Ulrich-Lai and Herman, 2009). Upon reaching the anterior pituitary, CRF stimulates the corticotropes to secrete adrenocorticotropic hormone (ACTH) into the systemic circulation (Tsigos and Chrousos, 2002; Denver, 2009). This hormone in turn acts on the inner adrenal cortex to synthesize and release glucocorticoids, such as cortisol (Denver, 2009; Ulrich-Lai and Herman, 2009). Circulating glucocorticoids induce a multitude of different responses which all act to promote energy mobilization; these include gluconeogenesis in the liver, liberation of amino acids, inhibition of glucose uptake into muscle and adipose tissue, increased lipolysis and suppression of immune and reproductive functions (Ulrich-Lai and Herman, 2009). Regulation of the system occurs through negative feedback of glucocorticoids at the hypothalamus and anterior pituitary, inhibiting the release of CRF and ACTH respectively; in addition, the action of glucocorticoids on neuronal inputs to the PVN restricts activation of the HPA axis (Tsigos and Chrousos, 2002; Denver, 2009). However, the HPA system, as it is understood in vertebrates, does not appear to be present in non-vertebrates (Lovejoy, 2009). Thus, given the evolutionary age of CRF-like peptides, it has become well ensconced in numerous physiological processes predating the HPA axis. However, CRF itself has a number of non-HPA associated functions including feeding, diuresis, metamorphosis, locomotion, and vocalization (Lovejoy and Balment, 1999). These regulatory actions may have evolved to accompany stress regulation. Given the widespread actions of the CRF family of peptides, evolutionarily older physiological systems may be working alongside the CRF lineage or interacting with elements of the CRF lineage in a neuromodulatory manner in complex organisms.

## Teneurin and the teneurin C-terminal associated peptides (TCAP)

The teneurins are a family of four type-II transmembrane proteins that are critical for morphogenesis and pattern formation in metazoans (Wang et al., 2005; Lovejoy et al., 2006, 2009; Tucker et al., 2007; Young and Leamey, 2009). Originally discovered in *Drosophila*, teneurins were later discovered in vertebrates to have four paralogs (teneurin-1 to -4) (Kenzelmann et al., 2007). Vertebrate teneurins are predominately expressed in the nervous system and have been implicated in neural development and maintenance (Lovejoy et al., 2006; Kenzelmann et al., 2007). They are typically about 2800 amino acids long with a structure that is highly conserved (Tucker et al., 2007). At the end of the C-terminus of teneurin-1 to -4 is a cleavable bioactive peptide termed the teneurin C-terminal-associated peptide (TCAP)-1 to -4. Recent studies, however, indicate that TCAP-1 is independently transcribed from teneurin-1, thus providing evidence for the independence of TCAPs from teneurins (Chand et al., 2012a).

**Figure 2:**
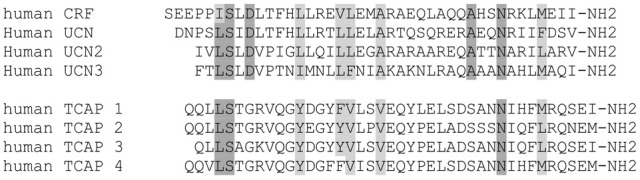
**Comparison of the primary structure between the TCAP and CRF families of peptides.** Amino acid identity among CRF paralogs are shown as dark gray, and continued among TCAP peptides where they match. Conservative homologous substitutions are shown in light gray. Note that the identity among TCAP peptides is much greater than that of CRF peptides.

Initial attempts to identify paralogous genes to the CRF family led to the discovery of this TCAP family (Qian et al., 2004). It was first discovered in a screening of the hypothalamic cDNA library of rainbow trout using a hamster urocortin probe (Barsyte et al., 1999; Qian et al., 2004). Sequence analyses revealed a putative peptide sequence on the 3′ region of teneurin-3 in trout and this peptide sequence was termed TCAP-3, by virtue of its association with teneurin-3 (Qian et al., 2004). Further analyses led to the discovery of the 3 other TCAP peptides on teneurins-1, -2, and -4 (Wang et al., 2005). The TCAP family is ubiquitously expressed among all metazoan species (Lovejoy et al., 2006; Tucker et al., 2012). The four vertebrate TCAP paralogs show about 80% identity with each other amongst metazoan species (Lovejoy et al., 2006, 2009). This high level of conservation indicates that this family of peptides evolved before the protostome-deuterostome divergence and that its role implicates a functional necessity for organisms. The teneurin-TCAP system likely evolved by horizontal gene transfer from a prokaryote gene to a choanoflagellate (Zhang et al., 2012), where subsequently this gene integrated within the choanoflagellate genome and was passed into subsequent metazoan speciation (Tucker et al., 2012; Chand et al., 2013; Tucker, 2013). Moreover, there is 13–30% similarity with various peptides and the CRF family of peptides (Wang et al., 2005; Lovejoy et al., 2006) (Figure 2). This level of sequence similarity, along with the peptide and mRNA size suggests that there may be a common origin in evolutionary history with the CRF peptides, although the evolutionary relationship between the lineages is not understood (Chand et al., 2013; de Lannoy and Lovejoy, in press).

The TCAP family of peptides all have characteristics of a cleavable bioactive peptide; a cleavage motif on the N-terminus and an amidation motif on the C-terminus. The TCAP-1, -2, and -4 peptides are 41-amino residues in length and TCAP-3 is 40 amino residues in length (Lovejoy et al., 2006). Of the four, mouse TCAP-1 is the only peptide in the family confirmed to be expressed as a separate transcript from the teneurin gene in adults, although this may be the case for mouse TCAP-3 as well. Thus, in our model of TCAP release, we assume that TCAP-2 and -4 are released by ectodomain cleavage, whereas TCAPs -1 and -3 may be released by ectodomain cleavage and by independent transcription, translation, and subsequent release via cytosolic processes. Studies in zebrafish, chick, and rodents indicate that although expressed throughout the brain, TCAP-1 is particularly concentrated in the limbic regions including the hippocampus, hypothalamus, and amygdala (Wang et al., 2005; Tan et al., 2009, 2011). It is in the rat hippocampus and amygdala that TCAP-1 has been shown to exert its neuromodulatory effects on behavior and stress.

## Neuromodulation of crf-induced behavior by TCAP-1

Gene expression studies on mouse brain revealed that TCAP-1 (as part of the teneurin-1 gene) was expressed mainly in the limbic system, hypothalamus and cerebellum (Wang et al., 2005), suggesting these areas as focal points of TCAP-1 action. However, like other neuromodulatory peptide systems, behavioral studies in rodents indicate that TCAP-1 has differential effects on anxiety depending upon the test, the treatment regimen, and perhaps even the baseline anxiety level of rats (Rotzinger et al., 2010). Acute administration of synthetic TCAP-1 into the BLA significantly increases the acoustic startle response in low-anxiety rats and decreases the response in high-anxiety rats (Wang et al., 2005). Repeated administration of TCAP-1 at picomole concentrations into the lateral ventricles decreased acoustic startle in all rats. A 50% decrease in the acoustic startle response was apparent 15 days after the last TCAP-1 treatment (Wang et al., 2005). Moreover, using the same regimen, TCAP-1 treatment completely ablated the CRF-induced increase in the acoustic startle response (Tan et al., 2008). In contrast, the same pre-treatment potentiated the effects of a CRF ICV challenge in the elevated plus maze and open field tests, where, upon CRF challenge, rats showed decreased open arm entries in the elevated plus maze, and decreased locomotion, as well as time, entries and distance traveled in the center zone of the open field (Tan et al., 2008). However, IV injections of TCAP-1 resulted in differential behaviors in response to an IV or ICV CRF challenge (Al Chawaf et al., 2007b). Overall, rats treated with repeated IV doses of TCAP-1, followed by an acute ICV CRF challenge were less anxious than rats given an injection of CRF via the IV route (Al Chawaf et al., 2007b). Again, these behavioral effects only occurred upon co-administration with CRF; TCAP alone had little effect on behavior, further indicating that this peptide plays a neuromodulatory role. This stress-mediated action of TCAP-1 is further reflected by studies using a cocaine reinstatement model (Kupferschmidt et al., 2010). Using the number of active lever responses as tests for reinstatement of cocaine seeking, prior TCAP-1 treatment in rodents resulted in fewer lever responses after a CRF injection. However, TCAP-1 pre-treatment did not modulate reinstatement by footshock stress, or cocaine-induced reinstatement.

The difference in effects of TCAP-1 on IV- vs. ICV- injected CRF in rats also suggests that TCAP-1 may be involved in the feedback of glucocorticoids to the brain. A previous study in mice (Martins et al., 1996) found that ICV-administered CRF induces its effects by targeting sites in the CNS as well as peripheral sites along the HPA axis. However, IV-administered CRF does not cross the blood–brain barrier and thus will only activate the HPA axis at the level of the pituitary and adrenal gland (Martins et al., 1996). Thus, the difference in TCAP-1 effects on ICV- and IV-administered CRF may be due to a modulatory effect of the glucocorticoid receptors or signaling in limbic regions by TCAP-1. In particular, TCAP-1 may be acting on the hippocampus and amygdala to change the sensitivity of the glucocorticoid feedback on the PVN CRF secretion.

Further studies indicated the action of TCAP-1 occurs at a pre-transcription and cellular activation level. TCAP-1 attenuates expression of CRF-induced *c-fos* in the amygdala (Tan et al., 2009). Rats pre-treated with TCAP-1 showed lower c-Fos immunoreactivity in the amygdala, medial prefrontal cortex, hippocampus, and the dorsal raphe nucleus upon ICV CRF injections. Activation of CRF promotes *c-fos* synthesis and expression through a PKA/cAMP/CamKII pathway that leads to CREB phosphorylation. Phosphorylated CREB then regulates *c-fos* gene transcription by binding to response elements CRE and AP-1 (Kovacs, 1998). Similarly to CRF, TCAP-1 leads to cAMP accumulation in vitro (Wang et al., 2005). Studies on Gn11 mouse immortalized cell lines show that TCAP-1 has a biphasic effect on cAMP such that low concentrations of TCAP-1 increased cAMP, and high concentrations decreased cAMP levels (Wang et al., 2005). Moreover, a CRF1 receptor antagonist addition failed to block the TCAP-1-induced increase in cAMP suggesting the presence of independent signaling pathways.

Administration of TCAP alone had a minor effect on *c-fos* activation *in vivo* in rats. However, TCAP administration after concomitant CRF administration produced significant behavioral changes (Tan et al., 2009). This strongly suggests that TCAP plays a neuromodulatory role on elements of CRF signaling systems. Evidence shows that glucocorticoids inhibit AP-1 and CRE responses (Kovacs, 1998). Thus, TCAP could regulate elements of CRF signaling through modulation of CRE and AP-1 activity. In addition, TCAP could inhibit CRF-induced *c-fos* through inhibition of CRF expression itself, as the promoter of the CRF gene contains a CRE (Hauger et al., 2006). Using luciferase reporter assays, we found that TCAP administration decreases AP-1 reporter activity in an immortalized N3 hypothalamic cell line (Nock et al., unpublished observations). Thus, TCAP's ability to attenuate CRF induced *c-fos* activation *in vivo* may be, in part, through interactions with the AP-1 response element. However, AP-1 is regulated by numerous elements, and TCAP-1 may regulate AP-1 activity by a CRF-independent mechanism.

*In vitro*, TCAP-1 administration did not alter CRF-induced cAMP increases in the hypothalamic cell line Gn11 (Wang et al., 2005), nor CRF-induced CRE activation in human embryonic kidney cells transfected with CRF_1_ and CRF_2_ receptors. *In vitro*, TCAP-1 does not modulate cellular distribution or total protein of CRF_1_, or the GC receptors GR and MR in mouse embryonic hippocampal E14 cells. Administration of TCAP-1 also did not modulate the phosphorylation of a CRF_1_ downstream transcription factor CREB. Repeated daily TCAP-1 administration in mice did not affect basal non-stressed HPA activity based on serum cortisol and ACTH levels. From these studies, it is apparent that under basal non-stressed conditions, TCAP-1 does not interact directly with HPA regulatory pathways involving CRF or GC receptors (De Almeida et al., unpublished studies).

The lack of direct interaction of TCAP-1 on CRF signal transduction systems led us to investigate alternative explanations for its mechanism. The similarity of the TCAP-1 primary structure to CRF and related peptides had led us to postulate that the peptide activated a G-protein coupled receptor (GPCR) similar to the family of GPCRs related to the CRF receptors. However,*in vitro* screening of most of the receptors in this family did not show any significant binding or activation (Lovejoy, unpublished studies). However, gene microarray profiling indicated a similarity to neurotrophic factor activation and a relationship with the dystroglycan complex (Chand et al., 2012b). Subsequent studies confirmed this relationship, thus TCAP-1 may be the first identified soluble ligand for the dystroglycan complex. The dystroglycan complex is hypothesized to be integral to the intracellular signaling of extracellular TCAP-1 due to a strong co-localization of TCAP-1 with β-dystroglycan, the plasma membrane bound subunit of the dystroglycan complex (Chand et al., 2012b). In essence, TCAP-1 mediates cytoskeletal re-organization in E14 cells by binding to the dystroglycan complex which then signals through a MEK- ERK1/2-dependent phosphorylation of stathmin and filamin A. This leads to f-actin polymerization, increased tubulin immunoreactivity, and increased filopodia development (Chand et al., 2012b). The ERK1/2 pathway is the proposed mechanism through which TCAP-1 modulates neurite and dendrite morphology in immortalized mouse hypothalamic cell lines, primary hippocampal cultures (Al Chawaf et al., 2007a), and Golgi-stained rat brains (Tan et al., 2011).

Within multiple areas of the mouse brain, CRF induces stress-related cytoskeletal changes. CRF_1_ activation in the CA3 mediates dendritic retraction by inducing destabilization of f-actin and the cell adhesion molecule nectin-3 (Chen et al., 2008; Wang et al., 2011). Activation of CRF_1_ in the locus coeruleus (LC) also mediates outgrowth of neuronal processes through the Rho GTPase Rac1 (Swinny and Valentino, 2006). In the hippocampus, this Rac1-dependent outgrowth of neuronal processes is counteracted by RhoA inhibition on dendritic spine formation and branching. Thus, TCAP-1 may exert its CRF modulating effects through countering CRF_1_ mediated changes in cytoskeletal re-organization-like synaptic plasticity within stress modulating brain areas that express both CRF_1_ and TCAP-1.

## Conclusion

Current data suggests that TCAP-1 acts as a separate and distinct peptide modulating system, which is involved in cytoskeletal-associated synaptic plasticity. From an evolutionary perspective, it is perhaps not surprising that TCAP-1 acts independently of CRF signaling. The teneurins and TCAP evolved early in metazoan history, whereas the CRF family of peptides and their role in integrating energy metabolism and the perception of stressors evolved later after evolution of vertebrates (Lovejoy and Jahan, 2006; Lovejoy, 2009). Moreover, the later evolution of the HPA axis in stress signaling and the independence of TCAP-1 neurmodulatory actions from the HPA axis further support a possible parallel evolution. The TCAP-1 peptide acts on the dystroglycan complex in order to signal intracellularly. This signaling may be downstream of other effectors from the CRF signaling pathway downstream of the CRF_1_ receptor. The observed TCAP-1 modulatory effects on CRF behaviors may then be mediated through TCAP-1 specific signaling that oppose CRF mediated effects on cellular cytoskeletal organization within brain areas that co-express TCAP-1 and CRF. This in turn manifests into modulation of CRF-mediated stress behaviors in brain regions that co-express CRF and TCAP-1.

### Conflict of interest statement

Dr. D. A. Lovejoy is a co-founder of Protagenic Therapeutics, Inc. The other authors declare that the research was conducted in the absence of any commercial or financial relationships that could be construed as a potential conflict of interest.

## References

[B1] Al Chawaf A. St. Amant K. Belsham D. Lovejoy D. A. (2007a). Regulation of neurite growth in immortalized mouse hypothalamic neurons and rat hippocampal primary cultures by teneurin C-terminal-associated peptide-1. Neuroscience 144, 1241–1254 10.1016/j.neuroscience.2006.09.062 17174479

[B2] Al Chawaf A. Xu K. Tan L. Vaccarino F. J. Lovejoy D. A. (2007b). Corticotropin-releasing factor (CRF)-induced behaviors are modulated by intravenous administration of teneurin C-terminal associated peptide-1 (TCAP-1). Peptides 28, 1406–1415 10.1016/j.peptides.2007.05.014 17644218

[B3] Barsyte D. Tipping D. R. Smart D. Conlon J. M. Baker B. I. Lovejoy D. A. (1999). Rainbow trout (*Oncorhynchus mykiss)* urotensin-I: structural differences between urotensins-I and urocortins. Gen. Comp. Endocrinol. 115, 169–177 10.1006/gcen.1999.7290 10417230

[B4] Chand D. Casatti C. A. de Lannoy L. Song L. Kollara A. Barsyte-Lovejoy D. (2012a). C-terminal processing of the teneurin proteins: independent actions of a teneurin C-terminal associated peptide in hippocampal cells. Mol. Cell. Neurosci. 52C, 38–50 10.1016/j.mcn.2012.09.006 23026563

[B5] Chand D. Song L. Delannoy L. Barsyte-Lovejoy D. Ackloo S. Boutros P. C. (2012b). C-terminal region of teneurin-1 co-localizes with dystroglycan and modulates cytoskeletal organization through an extracellular signal-related kinase-dependent stathmin- and filamin A-mediated mechanism in hippocampal cells. Neuroscience 219, 255–270 10.1016/j.neuroscience.2012.05.069 22698694

[B6] Chand D. de Lannoy L. Tucker R. Lovejoy D. A. (2013). Origin of chordate peptides by horizontal protozoan gene transfer in early metazoans and protists: evolution of the teneurin C-terminal associated peptides (TCAP). Gen. Comp. Endocrinol. 188, 144–150 10.1016/j.ygcen.2013.02.006 23453965

[B7] Chen Y. Dube C. M. Rice C. J. Baram T. Z. (2008). Rapid loss of dendritic spines after stress involves derangement of spine dynamics by corticotropin-releasing hormone. J. Neurosci. 28, 2903–2911 10.1523/JNEUROSCI.0225-08.2008 18337421 PMC2409370

[B8] de Lannoy L. Lovejoy D. A (in press). Evolution and phylogeny of the corticotropin-releasing factor (CRF) family of peptides: expansion and specialization in the vertebrates. J. Chem. Neuroanat. 24076419 10.1016/j.jchemneu.2013.09.006

[B9] Denver R. J. (2009). Structural and functional evolution of vertebrate neuroendocrine stress systems. Ann. N.Y. Acad. Sci. 1163, 1–16 10.1111/j.1749-6632.2009.04433.x 19456324

[B10] Donaldson C. J. Sutton S. W. Perrin M. P. Corrigan A. Z. Lewis K. A. Rivier J. E. (1996). Cloning and characterization of human urocortin. Endocrinology 137, 2167–2170 10.1210/en.137.5.2167 8612563

[B11] Hauger R. L. Risbrough V. Brauns O. Dautzenberg F. M. (2006). Corticotropin Releasing Factor (CRF) receptor signalling in the central nervous system: new molecular targets. CNS Neurol. Disord. Drug Targets 5, 453–479 10.2174/187152706777950684 16918397 PMC1925123

[B12] Kenzelmann D. Chiquet-Ehrismann R. Tucker R. P. (2007). Teneurins, a transmembrane protein family involved in cell communication during neuronal development. Cell. Mol. Life Sci. 64, 1452–1456 10.1007/s00018-007-7108-9 17502993 PMC11138457

[B13] Kovacs K. J. (1998). c-Fos as a transcription factor: a stressful (re)view from a functional map. Neurochem. Int. 33, 287–297 10.1016/S0197-0186(98)00023-0 9840219

[B14] Kupferschmidt D. A. Lovejoy D. A. Rotzinger S. Erb S. (2010). Teneurin C-terminal associated peptide-1 blocks the effects of corticotropin-releasing factor on reinstatement of cocaine seeking and on cocaine-induced behavioural sensitization. Br. J. Pharmacol. 162, 574–583 10.1111/j.1476-5381.2010.01055.x 20883474 PMC3041248

[B15] Lewis K. Li C. Perrin M. H. Blount A. Kunitake K. Donaldson C. (2001). Identification of urocortin III, an additional member of the corticotropin-releasing factor (CRF) family with high affinity for the CRF2 receptor. Proc. Natl. Acad. Sci. U.S.A. 98, 7570–7575 10.1073/pnas.121165198 11416224 PMC34709

[B16] Lovejoy D. A. (2009). Structural evolution of urotensin-I: retaining ancestral functions before corticotropin-releasing hormone evolution. Gen. Comp. Endocrinol. 164, 15–19 10.1016/j.ygcen.2009.04.014 19393654

[B17] Lovejoy D. A. Al Chawaf A. Cadinouche M. (2006). Teneurin C-terminal associated peptides: an enigmatic family of neuropeptides with structural similarity to the corticotrophin-releasing factor and calcitonin families of peptides. Gen. Comp. Endocrinol. 148, 299–305 10.1016/j.ygcen.2006.01.012 16524574

[B18] Lovejoy D. A. Balment R. J. (1999). Evolution and physiology of the corticotropin-releasing factor (CRF) family of neuropeptides in vertebrates. Gen. Comp. Endocrinol. 155, 1–22 10.1006/gcen.1999.7298 10375459

[B19] Lovejoy D. A. Jahan S. (2006). Phylogeny and evolution of the corticotropin releasing factor family of peptides. Gen. Comp. Endocrinol. 146, 1–8 10.1016/j.ygcen.2005.11.019 16472809

[B20] Lovejoy D. A. Rotzinger S. Barsyte-Lovejoy D. (2009). Evolution of complementary peptide systems: teneurin C-terminal-associated peptides and corticotropin-releasing factor superfamilies. Ann. N.Y. Acad. Sci. 1163, 215–220 10.1111/j.1749-6632.2008.03629.x 19456342

[B21] Martins J. M. Kastin A. J. Banks W. A. (1996). Unidirectional specific and modulated brain to blood transport of corticotropin-releasing hormone. Neuroendocrinology 63, 338–348 10.1159/000126974 8739889

[B22] Qian X. Barsyte-Lovejoy D. Wang L. Chewpoy B. Gautam N. Al Chawaf A. (2004). Cloning and characterization of teneurin C-terminal associated peptide (TCAP)-3 from the hypothalamus of an adult rainbow trout (*Oncorhynchus mykiss*). Gen. Comp. Endocrinol. 137, 205–216 10.1016/j.ygcen.2004.02.007 15158132

[B23] Reyes T. M. Lewis K. Perrin M. H. Kunitake K. S. Vaughan J. Arias C. A. (2001). Urocortin II: a member of the corticotropin-releasing factor (CRF) neuropeptide family that is selectively bound by type 2 CRF receptors. Proc. Natl. Acad. Sci. U.S.A. 98, 2843–2848 10.1073/pnas.051626398 11226328 PMC30227

[B24] Rotzinger S. Lovejoy D. A. Tan L. A. (2010). Behavioural effects of neuropeptides in rodent models of depression and anxiety. Peptides 31, 736–756 10.1016/j.peptides.2009.12.015 20026211

[B25] Swinny J. D. Valentino R. J. (2006). Corticotropin-releasing factor promotes growth of brain norepinephrine neuronal processes through Rho GTPase regulators of the actin cytoskeleton in rat. Eur. J. Neurosci. 24, 2481–2490 10.1111/j.1460-9568.2006.05129.x 17100837

[B26] Tan L. A. Al Chawaf A. Vaccarino F. J. Boutros P. C. Lovejoy D. A. (2011). Teneurin C-terminal associated peptide (TCAP)-1 modulates dendritic morphology in hippocampal neurons and decreases anxiety-like behaviors in rats. Physiol. Behav. 104, 199–204 10.1016/j.physbeh.2011.03.015 21411044

[B27] Tan L. A. Xu K. Vaccarino F. J. Lovejoy D. A. Rotzinger S. (2008). Repeated intracerebral teneurin C-terminal associated peptide (TCAP)-1 injections produce enduring changes in behavioral responses to corticotropin-releasing factor (CRF) in rat models of anxiety. Behav. Brain Res. 188, 195–200 10.1016/j.bbr.2007.10.032 18082275

[B28] Tan L. A. Xu K. Vaccarino F. J. Lovejoy D. A. Rotzinger S. (2009). Teneurin C-terminal associated peptide (TCAP)-1 attenuates corticotropin-releasing factor (CRF)-induced c-Fos expression in the limbic system and modulates anxiety behavior in male Wistar rats. Behav. Brain Res. 201, 198–206 10.1016/j.bbr.2009.02.013 19428634

[B29] Tsigos C. Chrousos G. P. (2002). Hypothalamic-pituitary-adrenal axis, neuroendocrine factors and stress. J. Psychosom. Res. 53, 865–871 10.1016/S0022-3999(02)00429-4 12377295

[B30] Tucker R. P. (2013). Horizontal gene transfer in choanoflagellates. J. Exp. Zoo. B Mol. Dev. Evol. 320, 1–9 10.1002/jez.b.22480 22997182

[B31] Tucker R. P. Beckmann J. Leachman N. T. Scholer J. Chiquet-Ehrismann R. (2012). Phylogenetic analysis of the teneurins: conserved features and premetazoan ancestry. Mol. Biol. Evol. 29, 1019–1029 10.1093/molbev/msr271 22045996 PMC3278476

[B32] Tucker R. P. Kenzelmann D. Trzebiatowska A. Chiquet-Ehrismann R. (2007). Teneurins: transmembrane proteins with fundamental roles in development. Int. J. Biochem. Cell Biol. 39, 292–297 10.1016/j.biocel.2006.09.012 17095284

[B33] Ulrich-Lai Y. M. Herman J. P. (2009). Neural regulation of endocrine and autonomic stress and responses. Nat. Rev. Neurosci. 10, 397–409 10.1038/nrn2647 19469025 PMC4240627

[B34] Vaughan J. Donaldson C. Bittencourt J. Perrin M. H. Lewis K. Sutton S. (1995). Urocortin, a mammalian neuropeptide related to fish urotensin-I and to corticotropin-releasing factor. Nature 378, 287–292 10.1038/378287a0 7477349

[B35] Wang L. Rotzinger S. Al Chawaf A. Elias C. F. Barsyte-Lovejoy D. Qian X. (2005). Teneurin proteins possess a carboxy terminal sequence with neuromodulatory activity. Mol. Brain Res. 133, 253–265 10.1016/j.molbrainres.2004.10.019 15710242

[B36] Wang X. D. Chen Y. Wolf M. Wagner K. V. Liebl C. (2011). Forebrain CRHR1 deficiency attenuates chronic stress-induced cognitive deficits and dendritic remodeling. Neurobiol. Dis. 42, 300–310 10.1016/j.nbd.2011.01.020 21296667 PMC3200197

[B37] Young T. R. Leamey C. A. (2009). Teneurins: important regulators of neural circuitry. Int. J. Biochem. Cell Biol. 41, 990–993 10.1016/j.biocel.2008.06.014 18723111

[B38] Zhang D. deSouza R. F. Anantharaman V. Iyer L. M. Aravind L. (2012). Polymorphic toxin systems: comprehensive characterization of trafficking modes, mechanism of action, immunity and ecology using comparative genomics. Biol. Direct. 7, 18 10.1186/1745-6150-7-18 22731697 PMC3482391

